# How Risk Factors Affect Head and Neck Squamous Cell Carcinoma (HNSCC) Tumor Immune Microenvironment (TIME): Their Influence on Immune Escape Mechanisms and Immunotherapy Strategy

**DOI:** 10.3390/biomedicines10102498

**Published:** 2022-10-07

**Authors:** Danilo Galizia, Silvia Minei, Elena Maldi, Giovanna Chilà, Alessio Polidori, Marco Carlo Merlano

**Affiliations:** 1 Candiolo Cancer Institute, FPO-IRCCS, 10060 Candiolo, Italy; 2 Post-Graduate School of Specialization in Medical Oncology, University of Bari ‘A. Moro’, 70120 Bari, Italy; 3 Division of Medical Oncology, A.O.U. Consorziale Policlinico di Bari, 70120 Bari, Italy

**Keywords:** head and neck, tumor microenvironment, smoking-associated, virus-associated

## Abstract

Most head and neck squamous cell carcinomas (HNSCCs) are caused by lifestyle, such as cigarette smoking, or by viruses, such as human papillomavirus (HPV) and Epstein–Barr virus (EBV). HNSCC remains a clinical challenge, notwithstanding the improvements observed in the past years, involving surgery, radiotherapy, and chemotherapy. Recurrent/metastatic (R/M) disease represents an unmet clinical need. Immunotherapy has improved the prognosis of a small proportion of these patients, but most still do not benefit. In the last decade, several preclinical and clinical studies have explored the HNSCC tumor immune microenvironment (TIME), identifying important differences between smoking-associated and virus-associated HNSCCs. This review aims to present how different etiologies affect the HNSCC TIME, affecting immune escape mechanisms and sensitivity to immunotherapy.

## 1. Introduction

Head and neck squamous cell cancer (HNSCC) represents the sixth most common cancer worldwide [1]. Two distinct HNSCC entities can be identified by their etiologies: the more frequent carcinogen-associated HNSCC, strongly related to tobacco and alcohol, and virus-associated HNSCC [2]. The well-known etiological risk factors for the latter are human papillomavirus (HPV—primarily type 16) and Epstein–Barr virus (EBV) [2,3,4].

In the last decade, immune checkpoint inhibitors (ICIs) have been approved by the FDA and EMA for (R/M) HNSCC not amenable to loco-regional treatment: nivolumab for second-line therapy in platinum-resistant patients, regardless of PD-L1 status [5,6], and pembrolizumab with or without chemotherapy in PD-L1-positive patients in the first-line setting [7,8]. Unfortunately, the impact of immunotherapy as a single approach on HNSCC remains marginal. Only 15–20% of patients achieve an objective clinical response [9].

## 2. HNSCC Tumor Microenvironment: General Considerations

In the last several years, clinical, genomic, and cellular studies have demonstrated that the HNSCC TIME is highly heterogeneous and immunosuppressive [10].

HNSCC can evade immune surveillance by deregulating key signaling steps for antitumor immunity, leading to unrestrained tumor growth [11]. This immunosuppressive mechanism includes a decrease in T-cell receptor (TCR) activity and HLA–peptide antigen interactions with the inactivation of the antigen-processing machinery, preventing the processing and presentation of tumor-associated antigens; other important immunosuppressive mechanisms are the upregulation of checkpoint inhibitory molecules, the induction of T-cell apoptosis, an increase in immunosuppressive Th2-type cytokines with augmented regulatory T-cell (Treg), myeloid-derived suppressor cell (MDSC), or M2 macrophage recruitment, and a decrease in chemokines that attract immune effector cells into the TIME [12,13]. Moreover, tumor cells are constrained by a dynamic process known as immunoediting, which, under selective pressure, favors the expansion of less immunogenic tumor cells able to escape the immune response and thus gain a survival advantage [14].

## 3. The Main Immunophenotypes Identify Specific Escape Mechanisms

Evaluating data from preclinical and translational research, Chen and Mellman [15] suggested clustering tumors into three major immune phenotypes: the inflamed phenotype (“hot”), the immune-excluded phenotype (“excluded”), and the immune-desert phenotype (“cold”).

The first profile is characterized by the presence of tumor-infiltrating lymphocytes in the tumor bed, together with myeloid cells and monocytic cells; the infiltrating immune cells and, in some cases, tumor cells exhibit checkpoint activation, i.e., programmed cell death protein 1 (PD-1) and its ligand (PD-L1). mRNA analysis performed on tumor sections is able to detect many proinflammatory and effector cytokines [16,17,18]. This immune phenotype may respond to ICIs, suggesting the presence of a pre-existing but inhibited antitumor immune response.

The second profile is the immune-excluded phenotype, which is characterized by a high presence of immune cells that do not infiltrate the tumor nests but remain trapped in the surrounding stroma [15,16,19,20]. After treatment with ICIs, CD8^+^ and CD4^+^ T cells can show evidence of activation and proliferation but not infiltration, suggesting a block in tumor homing. This tumor profile rarely responds to checkpoint inhibitors, suggesting that immune escape mechanisms are not related to the inhibitory effect of the PD-(L)1 axis [15].

**Figure 1:**
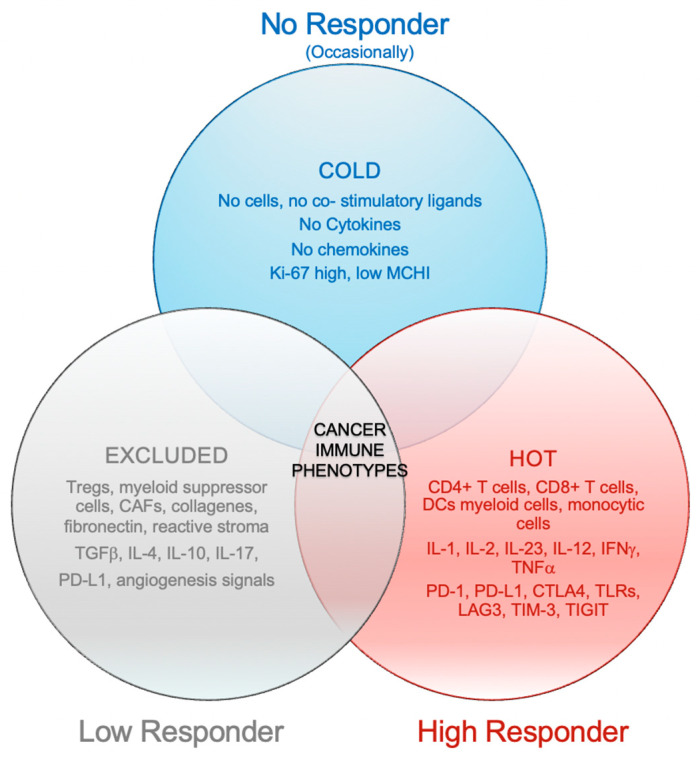
Cancer immune phenotypes.

The immune-desert phenotype is characterized by the rarity of T cells in either the tumor bed or the stroma [15,16,21,22] and the immunosuppressive reprogramming of the TIME with aberrant tumor vasculature and/or stroma. These tumors rarely respond to anti-PD-L1/PD-1 therapy [16]. This phenotype probably reflects the absence of pre-existing antitumor immunity, which suggests that the generation of tumor-specific T cells is the rate-limiting step. The “cold” and “excluded” phenotypes can both be considered non-inflamed tumors [15], as described in Figure 1.

Recently, a study that integrated genetic data with the RNA-seq-based deconvolution of immune cell populations and effector/regulatory molecules in a large cohort of HNSCC patients revealed that HNSCC is one of the most highly immune-infiltrated cancer types, with high NK and Treg cell presence; however, the authors identified a broad diversity in the levels of immune infiltration and activation across HNSCCs. In particular, tumors with the smoking-high signature have low levels of T-cell infiltration and activation [23].

Ribbat-Idel et al. [24] recently published a large comprehensive clinicopathological study on ICI-naive primary HNSCCs, aiming to categorize them as either immunologically “hot,” “cold,” or “excluded”, as presented above. Survival analysis showed significantly lower OS for “cold” primary HNSCC when compared to “hot” or “excluded” ones. In addition, they observed that the HPV association (as the p16 expression status) and the immune infiltration pattern were statistically significant independent factors for OS in multivariate analysis. The authors suggested considering this immunological classification as a prognosticator for HNSCC patients [24].

## 4. Different Microenvironments in HNSCC

Epidemiological studies have revealed a broad range of risk factors for HNSCC that can classify these tumors into two main groups: the first group, carcinogen-associated HNSCC, is related to tobacco consumption, alcohol consumption, and exposure to environmental pollutants, and the second group, virus-associated HNSCC, is related to HPV and EBV infections. Interestingly, several risk factors display geographical or cultural and/or habitual prevalence [25].

For instance, in regions such as Southeast Asia and Australia, HNSCC has a high prevalence associated with the consumption of specific carcinogen-containing products, such as betel-nut and tobacco chewing [25].

Below, we describe the differences between these two groups, focusing on the effect of these factors on the TIME.

### 4.1. Difference between Carcinogen-Associated and Virus-Associated HNSCC

Using multiplex immunohistochemistry, recent studies explored the HNSCC TIME and found that a myeloid-inflamed profile was associated with a poor prognosis and that high numbers of CD8^+^ T cells at the invasive margin of HPV-negative HNSCC were associated with prolonged overall survival, respectively [26,27]. 

Saloura et al. observed that HPV-positive tumors are enriched with CD8^+^ T cells and Tregs, and HPV-negative tumors show a lower abundance of CD8^+^ T cells but a high infiltration of M2 macrophages compared to HPV-positive tumors [28]. 

The difference in survival between HPV-positive and HPV-negative HNSCC patients could be due to an adaptive immune response directed against the viral antigens expressed by tumor cells, which determines a higher presence of tumor-infiltrating lymphocytes (TILs) and an inflamed gene expression profile [29]. A minority of HPV-positive oropharyngeal squamous cell carcinoma (OPSCC) patients respond poorly to treatment and have a dismal prognosis [30]. Smoking has been shown to reduce the survival benefit of individuals with HPV-positive oropharyngeal squamous cell carcinoma (OPSCC) [3], and therefore, it might be that these patients are also smokers. 

The disease-specific survival of OPSCC patients, stratified according to HPV status and tumor-infiltrating lymphocyte (TIL) levels, is lower in HPV-positive/low-TIL patients, and it is similar to that of HPV-negative patients [30]. This is consistent with the observation that not all HPV-positive OPSCCs are the same. 

EBV-positive nasopharyngeal carcinoma (NPC) displays an inflamed phenotype, according to Chen and Mellman [15]: immune cells are in close proximity to and in contact with NPC cells, instead of being embedded in the surrounding area away from the tumor core. Since the nasopharynx is one of the first defensive organs against viral and bacterial entry and infection, its microenvironment is physiologically highly reactive and immunogenic. In the normal nasopharyngeal stroma, two different major cell lineages are present: CD45+ immune cells, including T cells, B cells, NK cells, and MDSCs, as well as CD45- non-immune stromal cells, including fibroblasts and endothelial cells. The non-cancer-associated inflamed nasopharyngeal microenvironment differs from the TIME of NPC: the former shows an abundance of B cells, whereas T cells, NK cells, myeloid-derived cells, and fibroblasts are more likely to infiltrate the NPC TIME [31,32].

Finally, even if arising in similar anatomical sites, carcinogen-associated HNSCC and virus-associated HNSCC are characterized by distinct immune landscapes that strongly influence patients’ responses to immunotherapy and their outcomes, as described in Table 1. It is noteworthy that carcinogen-associated HPV-negative HNSCC displays a higher mutational load but low immune infiltration [23] compared to HPV-positive tumors; these factors influence the different clinical behaviors as well as the sensitivity to treatment and the prognosis. Exploring the distinct TIME features can help HNSCC investigators to rationally identify new immune targets and consequently plan new strategies for TIME-oriented clinical trials.

### 4.2. How Smoking Affects HNSCC TIME and Its Influence on Escape Mechanisms to Immunotherapy 

Carcinogen-associated HNSCC is usually diagnosed in men in their 50s or 60s, and it is strongly associated with smoking and alcohol; it is slowly declining globally, in part because of the decreased use of tobacco [2]. Even if treated with the best multimodal treatment (surgery, radiotherapy, and chemotherapy), locally advanced carcinogen-associated HNSCC still presents a dismal prognosis with a 40–50% 5-year OS [25]. 

Yet, in 2013, Hernandez C.P. et al. [33] demonstrated that cigarette smoke extract (CSE) is able to induce the inhibition of T-cell proliferation and activate T-cell apoptosis in vitro in a dose-dependent manner. Apoptosis enhanced by CSE was independent of caspase activation and endogenously mediated through reactive oxygen species (ROS) and reactive nitrogen species (RNS). This explanation is compelling and well supported by others: in fact, early studies have already shown that a chronically inflamed microenvironment (inflammatory disease or cancer-related) inhibits cytotoxic T cells and strengthens their hypofunction [34], for example, via NF-κB phosphorylation inhibition [35], a dimeric transcription factor involved in the expression of proteins necessary for innate immunity, apoptosis, and cell proliferation [36]. Moreover, the exposure of T cells to CSE induces the phosphorylation of eukaryotic translation initiation factor 2 alpha (eIF2), a factor involved in the expression of proteins promoting cellular apoptosis [33].

Besides in vitro studies, recently, de la Iglesia et al. [11] found that in an HPV-negative HNSCC population, active smoking led to an immunosuppressive signature, presenting as a decrease in cytotoxic T-cell tumor infiltration and the reduced expression of genes in the IFNα and IFNγ response pathways compared with former and never smokers. The smoking mutational signature, as found by TCGA, is correlated with tumor mutational burden (TMB) [23,37]; surprisingly, the authors analyzed the smoking status (using self-reporting) and TMB in a study subpopulation and did not find any correlations between these two parameters. Although the mutagenic effects of tobacco exposure are similar in HNSCC and squamous lung cancer (SLC), Desrichard A. et al. demonstrated an inverse correlation between the mutational smoking signature and the IFNγ signature in HNSCC patients and a positive correlation in SLC patients. Indeed, in HNSCC patients, the mutational smoking signature is associated with poorer survival, fewer tumor-infiltrating lymphocytes (TILs), and strong immunosuppressive effects. Conversely, in the SLC population, smoking is associated with a more inflamed tumor microenvironment, a higher TIL level, and a better response to immunotherapy [38]. In particular, HNSCC patients with a high mutational smoking signature show both low CD8^+^ T-cell infiltration and low IFNγ expression, suggesting that CD8^+^ T cells are not only less represented but also less capable of producing IFNγ [23]. In other words, smokers seem to have fewer infiltrating and also less functional CD8+ T cells.

Chemokine profile analyses performed in current smokers showed the decreased expression of the CXCL9,10,11/CXCR3 axis compared to current non-smokers. These chemokines are known to regulate immune cell migration, differentiation, and activation through the recruitment of cytotoxic lymphocytes and natural killer (NK) cells in response to IFNγ expression [39]. Recent data suggest that tumors associated with the IFNγ signature and inflamed phenotype have the highest probability of response and survival benefits when treated with anti-PD-1 checkpoint inhibitors [40,41]. An in vivo preclinical study showed the inhibition of anti-PD-1 inhibitor effects in CXCR3 knockout mice, indicating that the homing of T cells to the tumor through the CXCL9,10,11/CXCR3 axis may be critical for anti-PD-1 inhibitor efficacy [42]. 

Current smokers had significantly lower numbers of PD-L1-positive cells in the tumor core and tumor margins compared with never and former smokers [11], which is consistent with data that patients with smoking-high HNSCC have a lower response rate when given anti-PD-1 checkpoint inhibitors compared with smoking-low HNSCC [38]. Carcinogen-associated HNSCCs are characterized by enriched M2-phenotype macrophages, contributing to the creation of the immune-excluded TIME [43]. Monocytes recruited by specific cytokines released by the tumor (mainly CCL2 [44]) differentiate into M2-phenotype macrophages (M2s) in the hypoxic environment under VEGF pressure, losing their ability to migrate. Once they become residents in the TIME, M2 cells start to produce VEGF, which enhances the “feed-forward” loop, attracting new macrophages to the TIME, and TGF-β, one of the most potent immunosuppressive cytokines, which transforms normal fibroblasts and probably other stromal cells into cancer-associated fibroblasts (CAFs) [45]. CAFs, in turn, are known to release immunosuppressive cytokines (such as high levels of TGF-β, IL-10, and IL-6) in the TIME and to produce a stiff extracellular matrix that forms a sort of impenetrable barrier for immune cells and impairs oxygen and drug distribution [46]. In brief, the HNSCC TIME, ruled by high levels of TGF-β, leads to a self-renewing hypoxic environment. Brooks JM et al. [47] validated a combined hypoxia and immune prognostic classifier in HNSCC, finding three different categories: high hypoxia associated with low immune infiltration, low hypoxia associated with high immune infiltration and a mixed category. The first category is composed almost completely of carcinogen-associated HNSCCs with the worst overall survival in comparison to the other two. 

p53 is the most frequently mutated gene in carcinogen-associated HNSCC [48,49,50]. The loss of p53 function promotes the recruitment and instruction of suppressive myeloid CD11b^+^ cells, in part through the increased expression of CXCR3/CCR2-associated chemokines and macrophage-colony-stimulating factor (M-CSF), and attenuates CD4^+^ T-helper 1 (Th1) and CD8^+^ T-cell responses in vivo; additionally, p53-null tumors also show an accumulation of suppressive regulatory T (Treg) cells [10,51].

### 4.3. How HPV Affects HNSCC TIME and Influences Escape Mechanisms

HPV-associated OPSCC is increasing, mainly in the US and Western Europe, with a 10–30-year latency after oral sex exposure [9,25]. The outcome of non-metastatic HPV-positive oropharyngeal cancer is more favorable than the HPV-negative form, because it tends to have a better response to radiotherapy and chemotherapy, and patients are generally younger with better performance status [2]. 

Persistent infection with high-risk HPV (especially type 16) has been demonstrated to be the cause of virus-associated OPSCC. The virus exclusively infects basal keratinocytes and replicates only in fully mature epithelial cells, which are intrinsically programmed for death, and therefore, their death does not alert the immune system [52]. Hence, viral antigens are detectable only in superficial epithelial cells destined for desquamation and remote from immunological surveillance [53], enabling the virus to be undetected for long periods [54]. HPV-associated oncogenesis is controlled by the E6 and E7 oncoproteins [55]. The former promotes p53 degradation, upregulates telomerase activity, and maintains telomere integrity during repeated cell divisions, while E7 binds to retinoblastoma protein (pRb), allowing uncontrolled cell division. E7 can bind and degrade proteins that control cell-cycle entry in the basal and upper epithelial layers and thus is able to stimulate host genome instability through the deregulation of the centrosome cycle [56].

In previous clinical studies, the E6 and E7 long peptides showed their immunogenicity in being able to induce HPV-specific CD4^+^ and CD8^+^ T-cell proliferation and activity. From these findings, vaccines for the immunotherapy of HPV16-induced progressive infections, lesions, and malignancies have been developed [57].

Viral E6 and E7 oncoproteins are also known to be the main drivers of the immune escape mechanism in HPV-associated HNSCC [58], deregulating multiple immunity-related pathways to avoid recognition and clearance by the host immune system. E6 and E7 are able to downregulate activating chemokines such as CCL20, CCL2, and IL-8, leading to a reduction in dendritic cell (DC), monocyte, and neutrophil recruitment [59]; moreover, E6 and E7 enhance the release of inhibitory cytokines, such as: (a) IL-10, one of the most important immunosuppressive cytokines, which is able, among other functions, to initiate CD8^+^ cell exhaustion via activation of the transcriptional factor TOX, which contributes to the upregulation of PD1, TIGIT, TIM3, and LAG3 [60]; (b) TGF-β, which induces CD8^+^ T-cell and NK cell inhibition and the switch of CD4^+^ cells to CD4^+^ FOXP3^+^ (Tregs), eventually resulting in tumor tolerance and immune evasion [61]; (c) CXCL12, which contributes to Treg and Th2 cell recruitment [59]. 

The secretion of extracellular vesicles (EVs) is another important mechanism of immune escape [62] in HPV-positive cancers, based on cell–cell and cell–environment interactions between cancer and immune cells. It has been reported that HPV-positive cells release EVs that modify the microenvironment, enhancing tumor development and chemoresistance [58,63]. The role of EVs in the immune response was first described in 1996 [64]. In the last three decades, several studies have been conducted to explore EVs’ mechanism and influence on immune escape [65]. EVs harbor immunosuppressive molecules such as Fas-Ligand or tumor-necrosis-factor-related apoptosis-inducing ligand (TRAIL), checkpoint receptor ligands (PD-L1), or inhibitory cytokines (IL-10, TGF-β, and prostaglandin E2) [66]. 

E6 and E7 oncoproteins are also involved, in a dose-dependent manner, in interfering with the transcriptional activity of NF-KB [67], with a crucial negative switch on the inflammation triad, composed of IL-1 [68], TNF-α [69], and IL-6 [70].

Toll-like receptor 9 (TLR9) and stimulator of interferon genes (STING) are specific sensor proteins that are able to recognize DNA from viruses or bacteria within the cell cytosol or endosomal compartments and activate a type I IFN response [71]. Recently, Wang S et al. [72] showed that TLR9 was more often underexpressed in HPV-positive HNSCC tumors compared to their HPV-negative counterparts, and it is associated with a relatively poor prognosis. The HPV E7 oncoprotein can antagonize the STING pathway via NLRX1, which is a critical intermediary partner for STING turnover. In a preclinical model, the depletion of NLRX1 resulted in significantly improved type I IFN–dependent T-cell infiltration profiles and tumor control [73]. 

The TIME of HPV-positive HNSCC is considered “inflamed” by definition [43], enriched with CD8^+^ cells, CD4^+^ cells, Tregs, B cells, NKs, and M1-phenotype macrophages, with high expression of PD-L1 [10].

A recent study established a spectrum of differences between immune lineages in carcinogen-related versus HPV-positive HNSCC: besides CD8+ cells and Treg cells, similar lineages in both types of HNSCC, CD4^+^ T cells, B cells, and myeloid cells display different immune lineages, so it may require more tailored therapies [74]. These differences between HPV-positive and HPV-negative HNSCC TIMEs might be due to the presence of viral antigens (episomal or integrated components) [55], which may prime HPV-positive patients for enhanced antitumor immunity [74]. 

Using an RNA-seq analysis of 84 HPV-positive HNSCC tumors, Koneva et al. [55] explored the presence of HPV integration sites in cancer transcriptomes. They showed that integration-negative tumors (defined by the absence of the expression of viral–host fusion RNA transcripts) have better OS and higher levels of immune-related genes than those with integration-positive tumors [55]. Moreover, they found that the OS of integration-positive patients was similar to that of HPV-negative patients. Integration-negative tumors were characterized by strongly heightened signatures for immune cells, including CD4^+^, CD3^+^, regulatory, CD8^+^ T cells, NK cells, and B cells, compared with integration-positive tumors [55].

### 4.4. How EBV Affects Nasopharyngeal Cancer (NPC) TIME and Its Influence on Escape Mechanisms

Non-keratinized undifferentiated NPC is closely related to EBV infection [4]. Although this tumor originates from squamous cells of the nasopharyngeal mucosa, due to its outcome, it may be considered separately. NPC has a low incidence rate worldwide (just 0.7% of all cancers globally in 2018 [1]), but it is endemic in Southeast Asia with a high mortality rate [75]. Advanced disease contributes to high mortality rates in these endemic regions [75]. EBV infection in the epithelium of the nasopharynx can progress from lytic to latent infection, which is strongly associated with the carcinogenesis of NPC [76]. EBV is able to maintain the expression of various viral proteins, such as EBV nuclear antigen 1 (EBNA1), latent membrane protein 1 (LMP1), LMP2A, and LMP2B, during latent infection inside NPC cells [76]; all of these proteins are important in balancing viral replication and protein expression in order to prevent the presentation of viral antigens to the immune system [77], resulting in an oncogenic but weakly immunogenic nature.

The previously mentioned ethnic differences in NPC incidence suggest a major influence of genetic susceptibility, which is strongly linked to the immune escape mechanism underlying this disease. Epidemiological studies and recent large-scale genome-wide association studies have strongly demonstrated the association between HLA class I genes and NPC risk. Since HLA class I genes encode proteins that identify and present foreign antigens for the initiation of the host immune response against infected or malignant cells, it is hypothesized that high-risk populations with specific HLA haplotypes may be less efficient in mounting immune responses against latent EBV infection in the nasopharyngeal epithelium [78]. 

The knowledge of the role of EBV latent genes in immune evasion by NPC is yet to be completely achieved. Nevertheless, growing evidence shows several mechanisms that protect NPC cells from the host immune system. EBV-positive NPC cells are able to secrete cytokines and exosomes that drive the TIME toward immune suppression [79]: data from whole-exome sequencing and single-cell sequencing studies have progressively shown tumor infiltration by dysfunctional and exhausted CD8+ T cells and effector T (Teff) cells that overexpress inhibitory immune checkpoints, such as PD-L1, LAG3, galectin 9–TIM3, TIGIT, and CTLA4; moreover, other immunosuppressive cells, such as Tregs, TAMs-M2, and MDSCs, and various inhibitory cytokines have been identified to contribute to immunosuppression [79]. 

T-cell exhaustion represents one of the most important ways to block antitumor immune responses; unfortunately, the underlying mechanisms of this process are still largely unknown [80]. Recently, two different single-cell sequence analyses of CD8^+^ T cells from the TIME and the peripheral blood of EBV-positive NPC identified high numbers of exhausted CD8+ T cells, together with a significantly more restricted T-cell receptor (TCR) repertoire in both compartments, which explains the reduction in cytotoxic activity [32,81]. EBV-positive tumors are able to induce a highly variable pattern of TIME with increased numbers of different immune cell subsets, in particular, high frequencies of effector T cells, Tregs, and TAM-M2 cells. Of special interest, NPC cells can induce Tregs, suppress effector T cells, and regulate HLA class I expression, producing the so-called EBV-associated BamHI-C fragment rightward reading frame 1 (BCRF1) protein, which can encode viral IL-10 (vIL-10). vIL-10 has a very high sequence similarity to its human counterpart, IL-10 [82], exerting immunosuppressive effects on T cells but lacking the immunostimulatory effect of IL-10, which may contribute to the progression of tumors [83]. Furthermore, EBNA1 expression, for example, can upregulate CCL20, which recruits Treg cells that inhibit cytotoxic T-cell activities [84]. LMP1 can induce PD-L1- and galectin-9-containing exosomes, which enhance T-cell apoptosis and inhibit the functions of immune cells [85]. LMP2A and LMP2B are able to downregulate the antiviral response to interferon, inducing an increase in the turnover of interferon receptors [86]. Moreover, the abundant presence of a particular group of EBV-encoded microRNAs, named miR-BARTs, encoded by specific intronic regions of NPC, can activate the evasion of cell-surface major histocompatibility complex class I–related chain B for immune cell recognition, reducing the transcriptional activation of IFNγ and inhibiting NLRP3 inflammasome activation [87,88,89].

In NPC, the persistent activation of NF-kB pathways by somatic gene alterations or viral oncoproteins has been shown to play a crucial role in NPC tumorigenesis [90]. NF-kB is a family of five transcription factors: NF-kB1 (p105/p50, encoded by Nfkb1), NF-kB2 (p100/p52, Nfkb2), RelA (Rela), RelB (Relb), and c-Rel (Rel); in the resting state, NF-kB subunits are retained in the cytosol by IkB proteins and by unprocessed p105 and p100, which function as inhibitors. The activation of diverse receptors leads to the nuclear translocation of homo- or heterodimers of NF-kB subunits, which can then activate or repress gene transcription [91]. NF-kB signaling is key for immune function, and it is likely necessary for antitumor immunity [90]. Li YY et al. [92] showed that the majority of NPCs display the activation of the NF-kB signaling pathway as a result of somatic inactivating mutations in negative regulators of NF-kB. Previous studies have suggested a role for the non-canonical NF-kB pathway in Treg development and maintenance. Additionally, chronic inflammation recruits myeloid-derived suppressor cells (MDSCs), which promote NF-KB-controlled Treg cells to stimulate tumor angiogenesis and immune evasion [93]. In EBV-positive NPC cells, activated NF-kB regulates a number of chemokines (CXCL9, CXCL10, CX3CL1, and CCL20), which recruit tumor-infiltrating T lymphocytes and modulate the NPC tumor environment [78].

## 5. How Different HNSCC Risk Factors Influence the Response to Immunotherapy in Clinical Trials

There have been conflicting results on the response to immunotherapy in HPV-positive and HPV-negative patients from published HNSCC clinical trials (see Table 2). On the one hand, some clinical trials, such as KEYNOTE-012 [94,95] (pembrolizumab) and HAWK [96] (durvalumab), showed greater benefits and response rates in HPV-positive OPSCC patients compared to HPV-negative patients. On the other hand, the CheckMate 141 study [5,90] found an OS advantage in individuals treated with nivolumab compared to the investigator’s choice, irrespective of HPV status. However, patients positive for both HPV and PD-L1 expression presented the greatest OS benefit from nivolumab (HR 0.39; 95% CI 0.18–0.81). In KEYNOTE-040 [97], the median OS in the pembrolizumab cohort was 8.4 months (95% CI 6.4–9.4) vs. 6.9 months in the investigator’s cohort (95% CI 5.9–8.0). HPV-positive subjects had a similar OS benefit to the overall cohort in stratified analysis. More recently, KEYNOTE-048 [7] demonstrated that pembrolizumab +/− chemotherapy is superior to the standard first-line EXTREME regimen (cetuximab plus platinum-5-fluorouracil) in OS in a PD-L1 combined positive score (CPS) of ≥1 patient. As KEYNOTE-048 included both HPV-positive and HPV-negative patients, with a balanced proportion across arms, the new first-line standards of care for R/M HNSCC remain agnostic to HPV status. 

In an effort to better understand the impact of HPV status on the response to anti-PD1 immunotherapy, a meta-analysis has recently been published: Galvis MM et al. [98], assessing data collected from 11 clinical trials including 1860 R/M HNSCC patients treated with immunotherapy, reported that HPV-positive tumors were more responsive to immunotherapy than HPV-negative tumors for all outcome parameters they analyzed. Indeed, HPV-positive patients were numerically more likely to respond to immunotherapy than HPV-negative patients (risk ratio 1.29; 95 % CI = 0.85–1.96 I2 = 0%; overall effect *p* = 0.24). Moreover, when the authors simply compared the average OS of HPV-positive and HPV-negative tumors, they found that it was 11.5 months vs. 6.3 months, respectively (no statistical analysis reported) [98]. 

In 2021, a meta-analysis conducted on seven studies including 814 R/M HNSCC patients treated with PD-1 or PD-L1 inhibitors as single agents found that HPV-positive HNSCC patients displayed significantly longer OS than HPV-negative HNSCC patients. The objective response rate (ORR) of patients with HPV-positive HNSCC was significantly greater than that of their HPV-negative counterparts (OR = 1.77; 95% CI = 1.14–2.74; *p* = 0.01). Interestingly, the benefit was greater for the pooled anti-PD-L1 trials (OR = 2.66; 95% CI = 1.16–6.11; *p* = 0.02) compared to the pooled anti-PD-1 trials (OR = 1.51; 95% CI = 0.90–2.54; *p* = 0.12) [99]. The authors explained this last finding by the possibility that the blocking of PD-L1 on dendritic cells, abundant in the HPV-positive TIME, might relieve the cis sequestration of CD80 (this receptor can be induced by the HPV16 E7 oncoprotein), which allows the CD80/CD28 interaction to enhance T-cell priming [100] and, consequently, might represent a mechanism of the increased benefit in the HPV-positive population. 

Recently, a small but intriguing study analyzing circulating immune cells using flow cytometry and gene expression profiling in [24] HNSCC patients treated with ICIs showed that HPV-positive HNSCC had a higher content of plasma B cells and a more robust B-cell signature than HPV-negative HNSCC. In this limited series, elevated B-cell and plasma cell numbers were correlated with more favorable outcomes in terms of PFS. These findings, taking into account the small sample size of the study and its intrinsic limitations, suggest that B cells and plasma cells have beneficial roles in antitumor immunity within the TIME and are associated with favorable responses to immunotherapy [101]. 

Several clinical trials studied the use of ICIs in NPC; most of them were phase II trials of anti-PD-1 or anti-PD-L1 monotherapies in treatment-refractory populations (see Table 3). The results of these trials were pooled in a meta-analysis, which revealed an ORR of 27%, a 1-year PFS of 25%, and a 1-year OS of 61% for patients receiving anti-PD-1 antibodies [102].

The NCI-9742 phase II trial [103], which assessed the antitumor activity of nivolumab in R/M NPC patients who progressed to platinum-based chemotherapy, found that 9 out of 45 patients (ORR 20.5%) achieved an objective response after a median follow up of 12.5 months (1-year OS 59%). The authors did not identify any associations among PD-L1 expression, plasma EBV-DNA clearance, and survival.

KEYNOTE-028 [104] was a multicohort, nonrandomized, phase Ib trial that enrolled PD-L1-positive relapsed/metastatic NPC patients after chemotherapy progression. The results revealed that 7 of 27 patients (ORR 26%) achieved an objective response with pembrolizumab after a median follow-up of 20 months (1-year OS 63%). 

Significant results were achieved in a recent randomized, double-blind, phase 3 trial (CAPTAIN-1st) [105] comparing camrelizumab plus chemotherapy versus placebo plus chemotherapy as first-line treatment in patients with R/M NPC. The authors found that PFS was significantly prolonged in the experimental arm (9.7 months, 95% CI 8.3–11.4) compared to the placebo group (6.9 months, 5.9–7.3; HR 0.54, 95% CI 0.39–0.76; one-sided *p* = 0.0002). In a very recent real-world study enrolling 46 patients with R/M NPC treated with immunotherapy in two nonendemic regions, the ORR was 26.2%, and durable responses were observed. A low disease burden could serve as a biomarker for the response to ICIs.

Last year, a “Real-World” study carried out in two nonendemic regions [106] with 46 patients affected by R/M NPC and treated with ICIs showed an ORR of 26.2% with a median OS of 19.1 months (95%CI: 11.37−26.76) and a median PFS of 5.6 months (95% CI: 0.56−10.74). The researchers observed a negative association between aggressive disease features (i.e., more than three metastatic sites, metastatic disease at initial diagnosis, and positive pretreatment plasma EBV DNA) and the response to ICIs.

## 6. Conclusions

Immunotherapy constitutes an important weapon in medical oncologists’ arsenal against HNSCC. Unfortunately, currently approved immune checkpoint inhibitors (i.e., pembrolizumab and nivolumab) achieve positive clinical results in a minority of treated patients [6,7]. One way to overcome primary resistance to immune checkpoint inhibitors and enhance the therapeutic response consists of analyzing the HNSCC TIME, looking for new targets and/or immune axes to be pharmacologically modulated. Based on their etiology, carcinogen-associated HNSCC (mostly tobacco-associated) and virus-associated HPV-positive OPSCC or EBV-related NPC have distinct TIMEs, resulting in different immune escape mechanisms [13,23]. 

Active HNSCC smokers display an immunosuppressed TIME because of direct T-cell inhibition by CSE [33], the suppression of T-cell chemotaxis, and a consequent reduction in T-cell tumor infiltration [11]; moreover, the IFNα and IFNγ axis is downregulated, which, among other effects, leads to low PD-L1 and PD-L2 expression with a low response to immune checkpoint inhibitors [107]. One of the main factors that characterize the carcinogen-related HNSCC TIME and make it immunosuppressed and excluded is hypoxia [47]. Carcinogen-related HNSCC enhances VEGF production and consequently M2-cell migration and stabilization within the tumor, with TGF-β secretion [45], which promotes the epithelial–mesenchymal transition (EMT), angiogenesis, and cancer-associated fibroblast (CAF) activation, which in turn produces more TGF-β and angiogenic factors, leading to an immunosuppressive loop mechanism [108,109]. 

Although the HPV-associated OPSCC TIME is “hot” [43] because of the richness of activated immune cells, it can exploit viral E6 and E7 oncoproteins to overcome host immune system control [58]. In fact, E6 and E7 promote the release of inhibitory cytokines/chemokines, the downregulation of activating cytokines/chemokines, the secretion of immunosuppressive EVs, and the inhibition of an important pathway, namely, NF-KB, directly negatively affecting the inflammation triad [59,60,61]. Recently, E6 and E7 have been found to be involved in the inhibition of TLR9 and STING, external DNA sensor proteins, resulting in IFN immune-mediated response reduction [72,73]. 

Single ICIs still display marginal activity on advanced EBV-related NPC [79,110]. Better results are obtained when anti-PD1 treatment is associated with chemotherapy [105]. In NPC cells, a major immune escape mechanism is the existence of EBV in a state of type II latency, which limits the expression of non-coding RNAs and oncogenic EBV-related proteins, maintaining a low immunogenic profile, which favors evasion from host immune surveillance [79]. Although the NPC TIME is heavily infiltrated by immune cells around and within tumor lesions [111], it is characterized by the overexpression of inhibitory immune checkpoints, such as PD-L1, LAG3, galectin 9–TIM3, TIGIT, and CTLA4, which mark exhausted CD8+ T cells and, together with Tregs, M2s, MDSCs, and inhibitory cytokines, make it immunosuppressive. New attempts to better understand NPC TIME biology have already been performed in an attempt to identify specific NPC immune subsets using new-generation gene expression profiling techniques [112] and single-cell transcriptomics [111]. 

Taking into account all of the previously mentioned factors, our opinion is that HNSCC investigators should study virus-associated and carcinogen-associated HNSCC TIMEs separately. The differences between these two entities indicate that the exploration of immune cell infiltration starting from large omics datasets, the spatial interaction between tumor cells and immune cells, the CAFs in the stromal compartment, and all of the soluble factors (chemokines and cytokines) involved in the immune response and escape mechanism will define new clinical subsets, moving the focus from the cancer itself to its microenvironment. 

In particular, CAFs, activated by M2 cells via TGF-β, are able to upregulate the expression of several cytoskeletal regulators that remodel the extracellular matrix (ECM), increasing the global stiffness and creating a sort of wall between neoplastic cells and immune cells. This represents one of the main etiologies of the immune-excluded phenotype (see above) [113].

New preclinical and clinical findings will provide answers regarding the prognostic role of TIME subsets, help in defining patients who are most likely to respond to immunotherapy strategies, and create new research hypotheses about how to modulate the TIME in patients who seem to fit a less favorable profile. 

## Figures and Tables

**Table 1:** Factors influencing TIME: difference between carcinogen-associated and virus-associated HNSCC.

Factors Influencing TIME	Involvement in TIME	Carcinogen- Associated Tumors ^(ref)^	HPV- Associated Tumors ^(ref)^	EBV- Associated Tumors ^(ref)^
P53	Block of apoptosis and increased mutational charge	++ (48−50−51) ^exp^	+ (56) ^rev^	+ (88) ^exp^
LAG3 TIM3 TIGIT PD-1	T-cell exhaustion		+ (60, 66) ^rev^	++ (79) ^rev^
			+ (60, 66) ^rev^ + (60) ^rev^ + (60) ^rev^	++ (79) ^rev^ ++ (79) ^rev^ ++ (79) ^rev^
TMB	Increased tumor antigens	++ (37) ^exp^		
TCD8+	Infiltrate-positive	-- (23) ^exp^	++ (28) ^exp^	-- (84) ^cli^
TCD4+	Infiltrate-positive	-- (10, 51) ^rev, exp^	-- (55,60) ^rev c, rev^	
T regs	Infiltrate-negative	++ (10, 51) ^rev, exp^	++ (28) ^exp^	++ (85) ^exp^
TAM M2	Infiltrate-negative	++ (43) ^rev^	- (28) ^exp^	++ (93) ^rev^
NK cells	Infiltrate-positive	-- (23) ^exp^	-- (55) ^rev c^	+ (88) ^exp^
DC CD11b+	Infiltrate-positive Inhibition of antigen presentation	++ (10, 51) ^rev, exp^	-- (59) ^rev^	
IFNγ	Inflammatory response	-- (23) ^exp^	-- (71−73) ^rev^	-(71) ^rev^
CXCL9,10,11/ CXCR3 axis	Immune cell recruitment	- (42) ^exp^		++(78) ^rev^
CCL2 IL-8 CCL20	Proinflammatory Immunosuppression Treg recruitment	++ (44) ^rev^	-- (59) ^rev^ -- (59) ^rev^ -- (59) ^rev^	++ (84) ^cli^
TGF-β	Immunosuppression	++ (45,46) ^rev^	++ (61) ^rev^	
IL-10	Immunosuppression	++ (45,46) ^rev^	++ (60) ^rev^	++ (82) ^exp^
IL-6 CXCL12	Proinflammatory T-cell recruitment	++ (45,46) ^rev^	-- (70) ^rev^ + (59) ^rev^	

Legend: ++ more expressed/mutated; + expressed; - not expressed; -- downregulated; ^exp^: preclinical data; ^cli^: clinical data; ^rev^: review; ^rev c^: revision of clinical data.

**Table 2:** Clinical trials using ICIs in HNSCC.

Clinical Trial and Authors	Study Design	Setting	Intervention Drugs and ICIs	Population	ORR	PFS	OS
Keynote-012 [94] Seiwert, T.Y.; et al., *Lancet Oncol.* 2016	1b, nonrandomized, open-label	R/M HNSCC 2°line	Pembrolizumab	84 pts -23 (38%) HPV+ vs. 37 (62%) HPV- -61 (78%) PD-L1 > 1 -51 (85%) with tobacco use	18% (8 of 45; 95% CI 8–32); 25% in HPV + vs. 19% in HPV-	2 m (95% CI 2–4); 4 m in HPV+ and 2 m in HPV-	13 m (95% CI 5- not reached), not reached in HPV+ vs. 8 m in HPV-
Hawk Study [96] Zandberg, D.P. et al., *Eur J Cancer*, 2018	Phase II, single-arm	R/M HNSCC 2°line	Durvalumab	112 pts with PD-L1 > 25% -34 (34.3%) HPV+ vs. 65 (65.7%) HPV- -69 of 102 (61.8%) with tobacco use	16.2% (95% CI 9.9–24.4); 29.4% in HPV + vs. 10.8% in HPV-.	2.1 m (95% CI 1.9–3.7); 3.6 m in HPV+ and 1.8 m in HPV-	7.1 m (95% CI, 4.9–9.9); 10.2 m in HPV+ vs. 5 m in HPV-
CheckMate 141 [6] Ferris, R.L.; *Oral Oncol.* 2018	Phase III, randomized, open-label	R/M HNSCC 2°line	Nivolumab vs. standard care 2:1	240 pts received Nivolumab, and 121 received standard care -113 (26.2%) HPV+ in Nivolumab group and 65 (24.0%) in standard care -148 (57.3%) PD-L1 > 1 -191 (79.6%) with tobacco use in Nivolumab group and 85 (70.2%) in standard care	13.3% (95% CI, 9.3–18.3), including 6 CR and 26 PR, in Nivolumab group vs. 5.8% (95% CI, 2.4–11.6), including 1 CR and 6 PR, in standard care ORR in HPV+: 15.9% in Nivolumab group vs. 3.4% in standard care (OR: 5.28; 95% CI 0.64–43.4); ORR in HPV-: 8% in Nivolumab group vs. 11.1 in standard care (OR: 0.70; 95% CI 0.16–2.99) ORR in PD-L1 > 1: 17% in Nivolumab group vs. 1.6% in standard care (OR: 12.33; 95% CI 1.58–96.04)	2 m (95% CI, 1.9–2.1) in Nivolumab group vs. 2.3 m (95% CI, 1.9–3.1) in standard care	7.5 m (95% CI, 5.5–9.1) in Nivolumab group and 5.1 m (95% CI, 4–6) in standard care OS in HPV+: 9.1 m in Nivolumab group vs. 4.4 in standard care (HR: 0.56; 95% CI 0.35–0.99); OS in HPV-: 7.5 m in Nivolumab group vs. 5.8 in standard care (HR: 0.73; 95% CI 0.42–1.25, p: 0.55) OS in PD-L1 > 1: 8.7 m in Nivolumab group vs. 4.6 in standard care (HR: 0.55; 95% CI 0.36–0.83)
Keynote-040 [97] Cohen, E.E.W. et al. *Lancet* (2019)	Phase III, randomized, open-label	R/M HNSCC 2°line	Pembrolizumab vs. standard care	247 pts received Pembrolizumab, and 248 received standard care -61 (25%) HPV+ in Pembrolizumab group and 58 (23%) in standard care -196 (79%) PD-L1 CPS > 1 in Pembrolizumab group and 191 (77%) in standard care -179 (72%) with tobacco use in Pembrolizumab group and 182 (73%) in standard care	14.6 (95%CI 10.4–19.6) in Pembrolizumab group vs. 10.1% (95% CI 6.6–14.5) in standard care ORR in PD-L1 > 1: 17.3% in Pembrolizumab group vs. 9.9% in standard care	2.1 m (95%CI 2.1–2.3) in Pembrolizumab group vs. 2.3 m (95% CI 2.1–2.8) in standard care (HR: 0.96; 95% CI 0.79–1.16) PFS in PD-L1 CPS > 1; 2.2 m in Pembrolizumab group vs. 2.3 in standard care (HR: 0.86; 95% CI 0.69–1.06)	8.4 m (95%CI 6.4–9.4) in Pembrolizumab group vs. 6.9 m (95% CI 5.9–8.0) in standard care OS in PD-L1 CPS > 1; 8.7 m in Pembrolizumab group vs. 7.1 in standard care (HR: 0.74; 95% CI 0.58–0.93); OS in PD-L1 CPS > 50; 11.6 m in Pembrolizumab group vs. 6.6 in standard care (HR: 0.53; 95% CI 0.35–0.81)
Keynote-048 [7] Burtness, B. et al. *Lancet* (2019)	Phase III, randomized, open-label	R/M HNSCC 1°st line	Pembrolizumab alone vs. Pembrolizumab plus platinum and 5-FU vs. EXTREME	301 pts received Pembrolizumab alone, 281 pts received Pembrolizumab with chemotherapy, and 300 pts received EXTREME -63 (21%) HPV+ in Pembrolizumab-alone group, 60 (21%) in Pembrolizumab with chemotherapy group, and 66 (22%) in EXTREME group -257 (85%) PD-L1 CPS > 1 in Pembrolizumab-alone group, 242 (86%) in Pembrolizumab with chemotherapy group, and 255 (86%) in EXTREME group -239 (79%) with tobacco use in Pembrolizumab-alone group, 224 (80%) in Pembrolizumab with chemotherapy group, and 234 (78%) in EXTREME group	-16.9% in Pembrolizumab-alone group vs. 36% in EXTREME group in all pts -19.1% in Pembrolizumab-alone group vs. 34.9% in EXTREME group in CPS > 1 -23.3% in Pembrolizumab-alone group vs. 36.1% in EXTREME group in CPS > 20 (HR: 0.99; 95% CI 0.76–1.29) -35.6% in Pembrolizumab with chemo group vs. 36.3% m in EXTREME group in all pts -36.4% in Pembrolizumab with chemo group vs. 35.7% m in EXTREME group in CPS > 1 -42.9% in Pembrolizumab with chemo group vs. 38.2% m in EXTREME group in CPS > 20	-2.3 m in Pembrolizumab-alone group vs. 5.2 m in EXTREME group in all pts (HR: 1.29; 95% CI 1.09–1.53) -3.2 m in Pembrolizumab-alone group vs. 5.0 m in EXTREME group in CPS > 1 (HR: 1.13; 95% CI 0.94–1.36) -3.4 m in Pembrolizumab-alone group vs. 5.3 m in EXTREME group in CPS > 20 (HR: 0.99; 95% CI 0.76–1.29) -4.9 m in Pembrolizumab with chemo group vs. 5.2 m in EXTREME group in all pts (HR: 0.93; 95% CI 0.78–1.11) -5.1 m in Pembrolizumab with chemo group vs. 5.0 m in EXTREME group in CPS > 1 (HR: 0.84; 95% CI 0.69–1.02) -5.8 m in Pembrolizumab with chemo group vs. 5.3 m in EXTREME group in CPS > 20 (HR: 0.76; 95% CI 0.58–1.01)	-11.5 m in Pembrolizumab-alone group vs. 10.7 m in EXTREME group in all pts (HR: 0.83; 95% CI 0.70–0.99) -12.3 m in Pembrolizumab-alone group vs. 10.3 m in EXTREME group in CPS > 1 (HR: 0.74; 95% CI 0.61–0.90) -14.8 m in Pembrolizumab-alone group vs. 10.7 m in EXTREME group in CPS > 20 (HR: 0.58; 95% CI 0.44–0.78) -13.0 m in Pembrolizumab with chemo group vs. 10.7 m in EXTREME group in all pts (HR: 0.72; 95% CI 0.60–0.87) -14.7 m in Pembrolizumab with chemotherapy group vs. 11 m in EXTREME group in CPS > 20 (HR: 0.60; 95% CI 0.45–0.82) -13.6 m in Pembrolizumab with chemotherapy group vs. 10.4 m in EXTREME group in CPS > 1 (HR: 0.65; 95% CI 0.53–0.8)

**Table 3:** Clinical trials in NPC.

Clinical Trial and Authors	Study Design	Setting	Intervention: Drugs and ICIs	Population	Response	PFS	OS
NCI-9742 [103] Ma, B.B.Y. et al. *J. Clin. Oncol*. (2018)	Multicenter, phase II	R/M NPC previously treated	Nivolumab	45 pts received Nivolumab: -24 (53.3%) pts had PD-L1 in tumor cells < 1%, 18 pts (40%) had PD-L1 > 1, and 3 (6.7%) pts unknown -31 (68.9%) pts had PD-L1 in immune cells <1%, 10 pts (22.2%) had PD-L1 > 1, and 4 (8.9%) pts unknown -26 (57.8%) pts had HLA-A expression, 15 pts (33.3%) had HLA-A loss, and 4 (8.9%) pts unknown -21 (46.7%) pts had HLA-B expression, 20 pts (44.4%) had HLA-A loss, and 4 (8.9%) pts unknown	Trend of EBV DNA in cycle 1: not detectable in 1 (2.2%) pt, increasing in 19 (42.2%) pts, and decreasing in 25 (55.6%) pts. Confirmed response rate: 20.5% (95% CI 9.8–35.3): -CR in 1 (2.3%) pt, PR in 8 (18.2%) pts, SD in 15 (34.1%) pts, PD in 18 (40.9%) pts, and 2 (4.5%) pts NA 13% RR in 3 of 23 pts with PD-L1 tumor cells <1%, 29% RR in 2 of 7 pts with PD-L1 tumor cells <10%, and 33% RR in 4 of 11 pts with PD-L1 tumor cells > 10%	2.8 m (95% CI 1.8–7.4) PFS: -4.8 m (95% CI 2.7–14) in pts with loss of HLA-A and/or HLA-B -1.8 m (95% CI 1.7–7.4) in pts expressing HLA-A and/or HLA-B	17.1 m (95% CI, 10.9-NR) OS: -NR (95% CI 59.2%−96.8%) in pts with loss of HLA-A and/or HLA-B -10.9 m (95% CI 9.7-NR) in pts expressing HLA-A and/or HLA-B, *p* = 0.08
Keynote-028 [104] Hsu C. et al., *J. Clin. Oncolo*, (2017)	Phase 1b, nonrandomized, open-label	R/M NPC previously treated	Pembrolizumab	27 pts with PD-L1 > 1% received Pembrolizumab -10 (37%) pts had nonkeratinizing differentiated histology of NPC, 8 (29.6%) had undifferentiated histology, 6 (22.2%) had keratinizing squamous cells, and 3 (11.1%) were unclassified	25.9% (95% CI 11.1–46.3): -no CR 0% (95% CI, 0–12.8); PR in 7 (25.9%) pts, SD in 14 (51.9%) pts, and PD in 6 (22.2%) In 7 pts with PR, the DOR was 17.1 m (4.8 to > 22.1+1m), and all 7 of these pts had PD-L1 expression in the tumor only	6.5 m (95% CI, 3.6–13.4). 50% and 33.4% PFS rate at 6 and 12 months, respectively	16.5 m (95% CI, 10.1-NR) 85.2% and 63% OS rate at 6 and 12 months, respectively
CAPTAIN-1^ST^ [105] Yang, Y. et al. *Lancet Oncol*. (2021)	Multicenter, Phase III, randomized, double-blind	R/M NPC In 1°st line	Camrelizumab plus cis-gem (cam group) vs. placebo plus cis-gem (place group)	134 pts in cam group; 129 pts in place group - Positive baseline plasma EBV DNA level in 95 (71%) pts in cam group and 86 (67%) in place group; negative baseline plasma EBV DNA level in 39 (29%) in cam group and 43 (33%) in place group - Nonkeratinizing differentiated histology of NPC in 21 (16%) pts in cam group and 21 (16%) in place group, undifferentiated histology in 110 (82%) in cam group and 106 (82%) in place group, keratinizing squamous cells in 1 (<1%) in cam group and 1 (<1%) in place group, and other types in in 2 (1%) in cam group and 1 (>1%) in place group	87.3% (95% CI 80.5–92.4) in cam group and 80.6% (95% CI, 72.7–87.1) in place group -CR in 7 (5%) in cam group and 4 (3%) in place group, PR in 110 (82%) in cam group and 100 (78%) in place group, SD in 12 (9%) in cam group and 18 (14%) in place group, PD in 2 (1%) in cam group and 4 (3%) in place group, and NA in 3 (2%) in cam group and 3 (2%) in place group	9.7 m (95%, CI 8.3–11.4) in cam group vs. 6.9 m (95% CI, 5.9–7.3); HR: 0.54 (95% CI, 0.39–0.76) -9.9 m (95%, CI 8.1–12.3) in EBV DNA + cam group and 6.8 m (95%, CI 5.7–7.1) in EBV DNA+ place group -15.1 m (95%, CI 9.5-NR) in EBV DNA- cam group and 9.5 m (95%, CI 6.6–12.2) in EBV DNA- place group -11.4 m (95% CI 7-NR) in nonkeratinizing differentiated histology of NPC in cam group and 7.8 (95% CI 5.4–10.9) in place group, 10.2 m (95% CI 8.3–13.7) in undifferentiated histology in cam group and 6.9 m (95% CI 5.7–7.6) in place group, and 12.4 (95% CI 9.7–13.9) in other types in cam group and 7.1 (95% CI 5.8–8.3) in place group	NR in cam group and 22.6 m in place group (HR: 0.67, 95% CI 0.41–1.11)

## Data Availability

Not applicable.
